# Entering the Third Decade After Kidney Transplantation: Excellent Graft Function Refers to Superior Graft but Not Patient Survival

**DOI:** 10.3389/ti.2022.10675

**Published:** 2022-10-31

**Authors:** Anna Vera Reimann, Jakob Nilsson, Rudolf P. Wuethrich, Thomas F. Mueller, Thomas Schachtner

**Affiliations:** ^1^ Division of Nephrology, University Hospital Zurich, Zurich, Switzerland; ^2^ Department of Immunology, University Hospital Zurich, Zurich, Switzerland

**Keywords:** kidney transplantation, TCMR, *de novo* DSA, kidney allograft function, survival

## Abstract

Kidney transplant recipients (KTRs) with ultralong-term survival represent a growing, yet insufficiently studied patient cohort. In this single-center retrospective study, we analyzed 248 ultralong-term survivors (≥20 years). KTRs were classified into those with superior graft function (defined as eGFR ≥45 ml/min + proteinuria ≤300 mg/day + eGFR-slope ≤ 2 ml/min/1.73 m^2^/year) and inferior graft function regarding the risk of CKD progression. 20 years post-transplant, median eGFR was 54 ml/min (11–114), proteinuria 200 mg/24 h (0–7,620), eGFR decline 0.45 ml/min/1.73 m^2^/year (11.7 6.5) and DSA had been detected in 19.7% of KTRs. We identified 96 KTRs (38.7%) with superior (group 1) and 152 KTRs (61.3%) with inferior graft function (group 2). Donation after cardiac death, female sex, glomerulonephritis as primary disease, and early TCMR were independently associated with inferior graft function. Graft survival was significantly better in group 1 compared to group 2 (LogRank, *p* < 0.001). Besides group affiliation (HR 20.515, *p* = 0.003), multivariable analysis identified DSA development (HR 3.081, *p* = 0.023) and donor age (HR 1.032, *p* = 0.024) as independent factors. Interestingly, there was no significant difference in patient survival (LogRank, *p* = 0.350). In ultralong-term survivors, excellent graft function refers to superior graft survival but does not extend ultimate patient survival. DSA-formation should be taken seriously even in the ultralong-term.

## Graphical Abstract

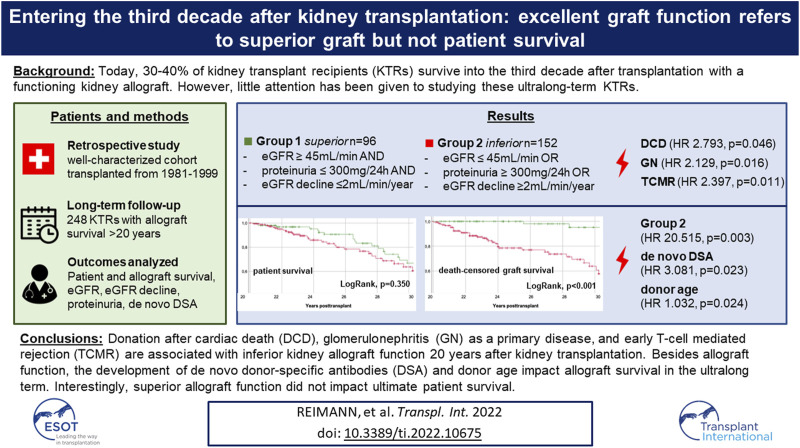

## Introduction

Kidney transplantation has become standard procedure in care of patients with end stage kidney disease (ESKD) and by today, is the preferred treatment for most of them (1). Over the past decades, short- and long-term graft survival have improved remarkably (2,3,4,5). For Europe, the Collaborative Transplant Study (CTS) reports an estimated 20-year death-uncensored graft survival rate of 41% for first deceased donor kidney transplant recipients (KTRs) from 1990–2020 and 16.8 years death-uncensored graft half-life (6). According to Coemans et al., who performed a comprehensive analysis of CTS data, death-censored 20-year graft survival rate even exceeded 50% for the transplant decade 1996–2005 (2). However, the authors reported survival data beyond 20 years to be sparse (2). The latest registry report from Australia and New Zealand (ANZDATA) reveals 30% 20-year death-uncensored graft survival for first deceased donor KTRs (3). Other comprehensive registry reports limit their analysis to a maximum of 10-year death-uncensored graft survival (for deceased donors 49.5% in the US (4), 58.5% in Canada (5).

Hence there is a growing population of KTRs who have lived with a functioning graft for several decades (7,8,9,10,11,12,13,14,15). Considering this development, surprisingly little attention has been given to the study of ultralong-term survivors (ULS) (7,8,9,10,11,12,13,14,15). Knowledge about their clinical characteristics, graft function, and alloimmunization is extremely limited and outcome as well as causes of graft losses in ULS have rarely been reported (7,8,9,10,11,12,13,14,15).

To optimize ultralong-term aftercare and to overcome the still important challenge of further improving long-term outcome (16), it is crucial to learn more about this particular patient group (9). To address these needs, we studied a large cohort of KTRs who have lived with a functioning graft for ≥20 years and aimed to investigate the following questions:

(1) What graft function (estimated glomerular filtration rate (eGFR), proteinuria, eGFR decline) do KTRs display 20 years post-transplant?
(2) What factors influence graft function 20 years post-transplant?
(3) What is the incidence of donor specific antibody (DSA)-formation in ULS?
(4) What is the outcome regarding graft and patient survival beyond 20 years post-transplant?
(5) What factors influence ultimate graft and patient survival of ULS?

## Methods

### KTRs and Data Collection

This single-center retrospective study was approved by the local Ethics committee of Zurich, Switzerland (Basec Number: 2019–02082) without informed consent requirement and performed in adherence to the declaration of Helsinki.

**FIGURE 1:**
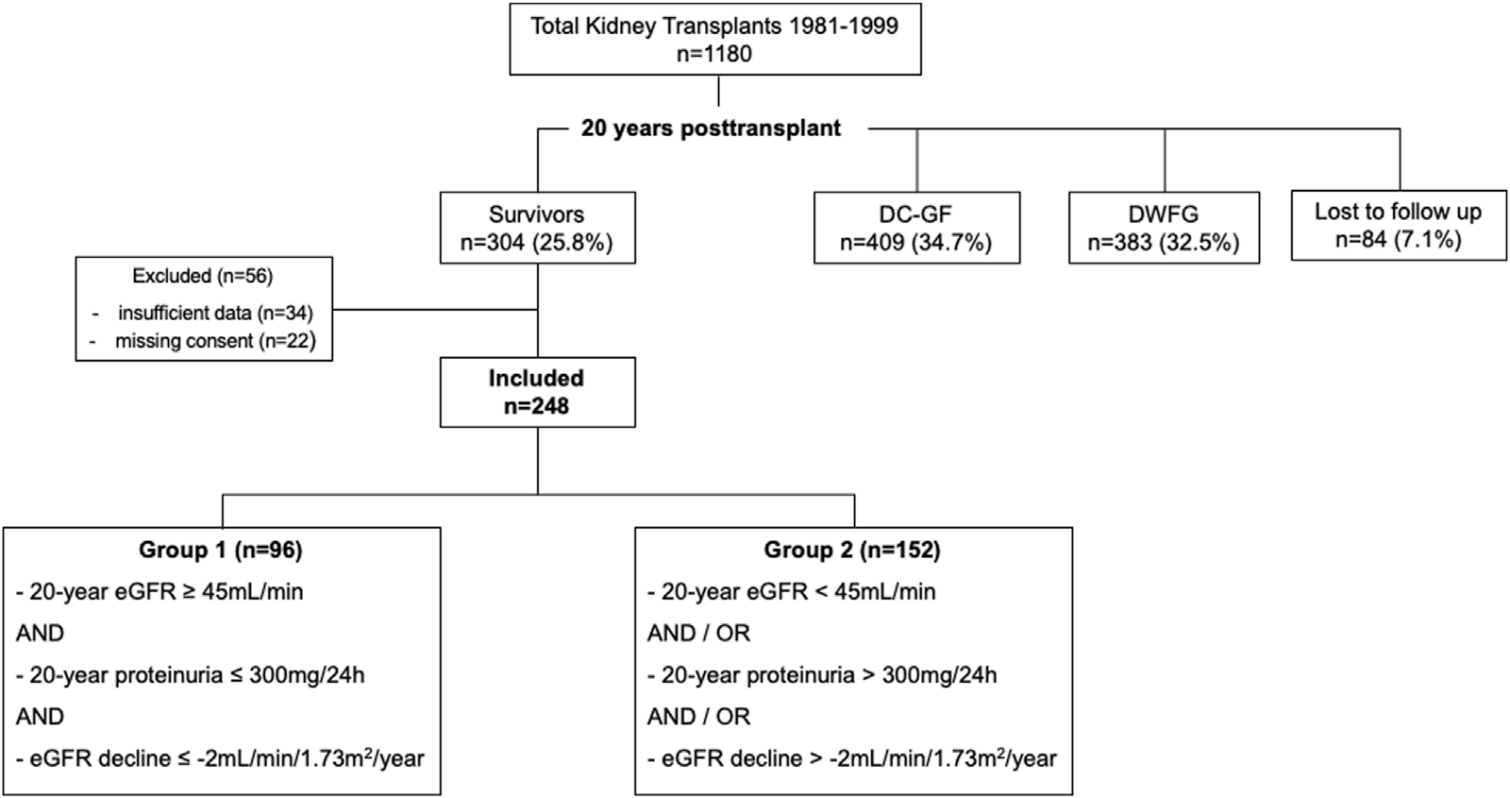
Flow chart all kidney transplants 1981–1999. DC-GF, death-censored graft failure. DWFG, death with functioning graft. Grouping criteria: 20-year eGFR: BSA-deindexed CKD-EPI at the 20-year *post-transplant visit*, 20-year proteinuria: Protein-to-creatinine ratio (mg/mmol) multiplied by 10 at the *2*0-year *post-transplant visit*. eGFR decline: eGFR CKD-EPI slope 15–20 years post-transplant.

We considered all adult (age ≥16 years at the date of transplantation) KTRs transplanted at University Hospital Zurich between 1 January 1981 and 31 December 1999. Among a total of 1,180 single-kidney transplantations performed at our institution during this era, we identified 304 KTRs with documented graft survival ≥20 years. 22 KTRs who had denied consent had to be excluded, further 34 KTRs due to insufficient data. This led to a total study cohort of 248 KTRs (Figure 1).

At our center, follow-up care after the first post-transplant year is generally performed quarterly in our outpatient clinic or, in stable conditions, by local nephrologists, complemented by an annual visit at our center. For data collection, we reviewed medical records from the electronic database of the hospital registry. End of follow-up and data collection was 31 January 2021. To evaluate characteristics 20 years post-transplant, we identified the 20-year post-transplant *visit* for each KTR*,* defined as the closest and most complete visit to the date of transplantation plus 20 years. For all KTRs, median time from transplantation to the 20-year post-transplant visit was 240 months (range 228–248 months). If the 20-year post-transplant visit did not reveal full data, we checked medical records from 19–21 years post-transplant for completion. Cases with insufficient documentation between 19–21 years post-transplant were excluded, as stated above.

### Graft Function

From serum-creatinine at the 1-year and 20-year post-transplant visit, we calculated baseline 1-year and 20-year eGFR, using the following formulas: Modification of Diet in Renal Disease (MDRD) (17), Chronic Kidney Disease Epidemiology Collaboration (CKD-EPI) (18), and Cockcroft-Gault (19). We did not include the race coefficient for MDRD and CKD-EPI (20). For MDRD and CKD-EPI, we additionally calculated body surface area (BSA)-deindexed eGFR, multiplying eGFR by KTR’s individual BSA (21), divided by 1.73 m^2^ (22,23). To indicate stability or decline of graft function, respectively, eGFR (CKD-EPI) slopes (24) were calculated for the last 5 years of the 20-year period, i.e., 15–20 years post-transplant. Baseline 20-year proteinuria was assessed by multiplying urine-to-creatinine ratio (PCR) (mg/mmol) from spot urine at the 20-year post-transplant visit by 10 (25). For 31 KTRs (12.5%), PCR was calculated from 24-hour collection urine, as before 2005, measurement of proteinuria was obtained by 24-hour collection urine at our center. PCR below detection limit was included in the analysis with a value of zero.

### Maintenance Immunosuppression and DSA-Screening

All donors and recipients were typed for human leukocyte antigen (HLA)-A, -B and -DR. Since approximately 2009, annual HLA antibody-monitoring using Luminex based assay (One Lambda, Canoga Park, CA, United States) became standard procedure in KTR-care at our center. In case of worsening graft function or progression of proteinuria, screening may have been performed more often. If Luminex mix assay was positive and/or clinical suspicion was high, an additional Luminex single antigen bead assay was performed to test for DSA. We classified KTRs with ≥1 DSA-positive Luminex single antigen bead assay up to 21 years post-transplant as DSA-positive, irrespective of the level of mean fluorescence intensity (MFI). KTRs were classified as DSA-negative in case HLA antibody-monitoring (Luminex mix assay only or both, Luminex mix and single bead assay) did not show DSA up to 21 years post-transplant or if the very first screening was performed beyond 21 years post-transplant and negative for DSA*.* KTRs were excluded from this sub-analysis in case of missing HLA antibody-screening during the observation period (*n* = 36, 14.5%) or if the very first screening was performed beyond 21 years post-transplant and DSA-positive, thus the date of DSA-occurrence was indeterminable (*n* = 14, 5.6%).

### Group Categorization

According to the KDIGO 2012 Clinical Practice Guideline for the risk of CKD progression, KTRs were stratified into two groups based on graft function 20 years post-transplant. (50) Criteria for superior graft function (Group 1) were: 1) 20-year eGFR ≥45 ml/min (BSA-deindexed CKD-EPI), 2) 20-year proteinuria ≤300 mg/24 h, and 3) eGFR (CKD-EPI) decline ≤-2 ml/min/1.73 m^2^/year 15–20 years post-transplant. Subjects in group 1 had to meet all 3 criteria. KTRs who did not pass ≥1 criteria were assigned to group 2. Two cases with missing data on proteinuria that fulfilled the other criteria for group 1 were classified as insufficient data and excluded, consequently. Two cases were categorized according to BSA-indexed CKD-EPI, because of unknown BSA (missing documentation of patient’s height).

### Survival

We separately studied patient survival (treating graft loss as a censored event), death-uncensored and death-censored (treating death as a censored event) graft survival, calculated from the date of transplantation to KTR’s death or graft loss (return to permanent dialysis or re-transplantation), whatever came first. If there was no event, survival dates were censored at the date of last follow-up or end of data collection (31 January 2021).

Specific causes of graft loss and results of indication biopsy were evaluated for KTRs with death-censored graft loss in group 1.

### Calculation of Predicted Indirectly ReCognizable HLA-Epitopes Scores

The HLA-derived mismatched peptide epitopes presented by KTRs HLA-molecules were calculated using the PIRCHE-II algorithm. Presentation of both HLA class I (HLA-A, B) and HLA class II derived peptides (HLA-DR, DQ) were calculated for each HLA locus. Detection of HLA antigens was performed by DNA-based HLA-typing technology using blood samples. Either sequence-specific oligonucleotide (SSO) or sequence-specific primer (SSP) technologies were used to generate low-resolution HLA typing results. The imputation of probable allele resolution results needed for the PIRCHE-II calculation was achieved by the use of the imputation algorithm included in the PIRCHE-II calculation. The PIRCHE-II algorithm is available online (https://www.PIRCHE-II.org).

### Statistical Analysis

Statistical analysis was performed using SPSS (Version 26, IBM, Armonk, NY, United States). Continuous variables are expressed as median (range minimum-maximum) and compared using Mann Whitney-U Test. Categorical data are expressed as number (%) and compared using Chi (2) test, corrected for Yates in 2x2 tables. If expected cell count was ≤5, we used Fisher’s Exact test instead. Missing values were not imputed. Survival was analysed using the Kaplan-Meier method and compared with LogRank test. Univariable and multivariable Cox proportional hazards models with enter method were used to investigate factors associated with survival. Variables with a *p*-value ≤0.05 in the univariable analysis were included in the multivariable model. For categorical variables in the multivariable model, assumption of proportional hazards was assessed visually by Kaplan Meier curves (26). For all tests, statistical significance was assumed for a two-tailed *p*-value <0.05.

## Results

Table 1 shows basic characteristics, Table 2 post-transplant complications and 1-year graft function, and Table 3 multivariable Cox regression analysis for group categorization of the 248 KTRs included in this study. Median KTR-age at the date of transplantation was 39.9 years, 92/248 (37.1%) KTRs were female.

**TABLE 1:** Basic recipient and donor characteristics.

20-year survivors	Total (*n* = 248)		Group 1 (*n* = 96)		Group 2 (*n* = 152)		*p*-value
	n		n		n		
**First transplant**	248	227 (91.5%)	96	92 (95.8%)	152	135 (88%)	
Second transplant		21 (8.5%)		4 (4.2%)		17 (11.2%)	0.089
**Female KTR**	248	92 (37.1%)	96	26 (27.1%)	152	66 (43.4%)	0.014*
**KTR age (years)** ^**a**^	248	39.9 (17.3–68.8)	96	38.1 (17.6–67.3)	152	40.3 (17.3–68.8)	0.663
**Cause of ESKD** ^**b**^	248		96		152		
GN^c^		89 (35.9%)		28 (29.2%)		61 (40.1%)	0.106
Uropathy^d^		45 (18.1%)		16 (16.7%)		29 (19.1%)	0.756
Diabetes mellitus		5 (2.0%)		2 (2.1%)		3 (2.0%)	1.0
Hypertension		4 (1.6%)		1 (1.0%)		3 (2.0%)	1.0
ADPKD^e^		30 (12.1%)		13 (13.5%)		17 (11.2%)	0.723
Alport syndrome		9 (3.6%)		6 (6.3%)		3 (2.0%)	0.093
Other		28 (11.3%)		12 (12.5%)		16 (10.5%)	0.785
Unknown		38 (15.3%)		18 (18.8%)		20 (13.2%)	0.313
**Pretransplant dialysis**	248		96		152		
preemptive transplantation		6 (2.4%)		2 (2.1%)		4 (2.6%)	1.0
HD^f^ (only HD or PD/HD)		193 (77.8%)		77 (80.2%)		116 (76.3%)	
only PD^g^		49 (19.8%)		17 (17.7%)		32 (21.1%)	0.615
pretransplant dialysis (months)^h^	221	25 (2–164)	90	22 (2–120)	131	28 (3–164)	0.047*
**Total HLA Mismatch (A, B, DR)**	248	3 (0–6)	96	3 (1–6)	152	3 (0–6)	0.343
**Total PIRCHE-II (A, B, DR)**		38.23 (0–111.63)		38.99 (14.07–97.72)		37.92 (0–111.63)	0.663
0–2 HLA Mismatches		48 (19.4%)		15 (15.6%)		33 (21.7%)	
3–6 HLA Mismatches		200 (80.6%)		81 (84.4%)		119 (78.3%)	0.309
HLA A Mismatch		1 (0–2)		1 (0–2)		1 (0–2)	0.07
HLA B Mismatch		1 (0–2)		1 (0–2)		1 (0–2)	0.712
PIRCHE-II (A, B)		26.35 (0–85.74)		28.13 (4.31–79.97)		25.24 (0–85.74)	0.665
HLA DR Mismatch		1 (0–2)		1 (0–2)		1 (0–2)	0.813
PIRCHE-II (DR)		8.06 (0–58.58)		6.94 (0–38.70)		9.44 (0–58.58)	0.221
**Donor characteristics**							
Living donor transplant	248	14 (5.6%)	96	3 (3.1%)	152	11 (7.2%)	0.278
Donation after cardiac death	248	28 (11.3%)	96	6 (6.3%)	152	22 (14.5%)	0.063
Donor age (years)	247	32 (3–72)	96	25 (3–63)	151	37 (3–72)	0.001**
Male donor	245	167 (68.2%)	94	65 (69.1%)	151	102 (67.5%)	0.904
CIT (hours)^i^	242	14 (1–34)	96	14.25 (1.5–34)	146	13.5 (1–32.5)	0.307
**Era of transplantation**	248		96		152		
1981–1989		92 (37.1%)		41 (42.7%)		51 (33.6%)	
1990–1999		156 (62.9%)		55 (57.3%)		101 (66.4%)	0.187

^a^ At the date of transplantation.

^b^ End stage kidney disease.

^c^ Glomerulonephritis, incl. vasculitis, systemic lupus erythematosus, and suspected chronic GN.

^d^ Incl. congenital anomalies of the kidney and urinary tract, CAKUT.

^e^ Autosomal dominant polycystic kidney disease.

^f^ Hemodialysis (only HD or both, PD and HD).

^g^ Peritoneal dialysis.

^h^ Only KTRs with the first transplant.

^i^ Cold ischemia time.

**TABLE 2:** Post-transplant complications and 1-year kidney allograft function.

8-year survivors	Total (*n* = 248)		Group 1 (*n* = 96)		Group 2 (*n* = 152)		*p*-value
	n		n			n	
Delayed graft function (DGF)	248	37 (14.9%)	96	10 (10.4%)	152	27 (17.8%)	0.143
**Rejection**	248	80 (32.3%)	96	20 (20.8%)	152	60 (39.5%)	0.002*
Early TCMR (<12 months)	248	53 (21.4%)	96	17 (17.8%)	152	46 (30.3%)	0.036*
Late TCMR (>12 months)	248	3 (1.2%)	96	0 (0.0%)	152	3 (2.0%)	0.285
Late ABMR (>12 months)	248	14 (5.6%)	96	3 (1.2%)	152	11 (7.2%)	0.259
Early CMV infection (<12 months)	248	25 (10.1%)	96	8 (8.3%)	152	17 (11.2%)	0.523
Post-transplant parathyreoidectomy	248	20 (8.1%)	96	5 (5.2%)	152	15 (9.9%)	0.235
**Graft function at 1 year post-transplantation**							
Serum-creatinine µmol/L	215	96 (48–145)	86	98 (48–138)	139	95 (58–145)	0.558
eGFR CKD-EPI^a^	215	70 (43–117)	86	70 (43–115)	139	72 (45–117)	0.498

^a^ ml/min/1.73 m^2^.

**TABLE 3:** Cox Regression analysis to assess group classification of KTRs 20 years post-transplantation.

Multivariate Cox regression analysis	HR	95% CI	*p*-Value
Number of transplants (second)	1.385	0.287–6.691	0.685
Recipient sex (female)	2.473	1.329–4.604	0.004*
Cause of ESKD (GN)	2.129	1.152–3.934	0.016*
Pretransplant dialysis (months)	1.015	1.001–1.030	0.041*
Donation after cardiac death (DCD)	2.793	1.017–7.667	0.046*
Donor age (years)	1.037	1.017–1.058	<0.001*
Early TCMR (<12 months)	2.397	1.222–4.700	0.011*

Multivariable Cox regression models for group classification at 20 years post-transplantation. Reference category in parentheses. HR, hazard ratio; CI, confidence interval.

### Graft Function

Table 4 shows detailed information on graft function 20 years post-transplant. Median serum-creatinine was 124 μmol/L, median eGFR 54 ml/min (BSA-deindexed CKD-EPI), median proteinuria 200 mg/24 h, and median eGFR decline −0.45 ml/min/1.73 m^2^/year. CKD-related laboratory findings are shown in the Supplementary Table S1.

**TABLE 4:** Characteristics and graft function 20 years posttransplant.

20-year survivors	Total (*n* = 248)		Group 1 (n = 96)		Group 2 (*n* = 152)		*p*-value
	N		n		n		
**KTR age (years)** ^**a**^	248	59.9 (37.1–89.1)	96	58.2 (37.9–87.4)	152	60.4 (37.1–89.1)	0.643
**BMI (kg/m** ^**2**^ **)** ^**b**^	246	25.2 (14–40.8)	95	25.5 (18.1–40.8)	151	24.7 (14–38.9)	0.291
BMI <18.5 kg/m^2^		4 (1.6%)		1 (1.1%)		3 (2.0%)	
BMI 18.5–24.9 kg/m^2^		116 (47.2%)		41(43.2%)		75 (49.7%)	
BMI 25–29.9 kg/m^2^		86 (35.0%)		37 (38.9%)		49 (32.5%)	
BMI 30–34.9 kg/m^2^		29 (11.8%)		12 (12.6%)		17 (11.3%)	
BMI ≥35 kg/m^2^		11 (4.5%)		4 (4.2%)		7 (4.6%)	0.814
**Graft function**	248		96		152		
Serum-creatinine µmol/L		124 (54–496)		101 (54–170)		142 (60–496)	<0.001***
eGFR CKD-EPI^c^		51 (11–102)		63 (40–98)		41 (11–102)	<0.001***
eGFR deindexed CKD-EPI^d^		54 (11–114)		65 (45–114)		43 (11–111)	<0.001***
eGFR MDRD^c^		48 (11–97)		59 (38–97)		39 (11–92)	<0.001***
eGFR deindexed MDRD^d^		51 (12–104)		62 (43–104)		41 (12–103)	<0.001***
eGFR Cockcroft Gault^d^		55 (11–140)		67 (34–117)		45 (11–140)	<0.001***
**CKD stage** ^**e**^	248		96		152		
G1 (eGFR ≥90 ml/min)		13 (5.2%)		7 (7.3%)		6 (3.9%)	
G2 (eGFR 60–89 ml/min)		77 (31.0%)		55 (57.3%)		22 (14.5%)	
G3a (eGFR 45–59 ml/min)		70 (28.2%)		34 (35.4%)		36 (23.7%)	
G3b (eGFR 30–44 ml/min)		52 (21.0%)		0(0.0%)		52 (34.2%)	
G4 (eGFR 15–29 ml/min)		33 (13.3%)		0 (0.0%)		33 (21.7%)	
G5 (eGFR <15 ml/min)		3 (1.2%)		0 (0.0%)		3 (2.0%)	<0.001***
**Proteinuria** ^**f**^	246	200 (0–7,620)	96	98 (0–300)	150	400 (0–7,620)	<0.001***
0–300 mg/24 h		152 (61.8%)		96 (100%)		56 (37.3%)	
301–1,000 mg/24 h		67 (27.2%)		0 (0.0%)		67 (44.7%)	
1,001–3,500 mg/24 h		21 (8.5%)		0 (0.0%)		21 (14.0%)	
>3,500 mg/24 h		6 (2.4%)		0 (0.0%)		6 (4.0%)	<0.001***
**eGFR decline** ^**g**^	246	−0.45 (−11.7–6.5)	96	0.45(−2.0–6.5)	150	−1.25 (−11.7–6.3)	<0.001***
≤ −2 ml/min/1.73 m^2^/year		189 (76.8%)		96 (100%)		93 (62.0%)	
> −2 ml/min/1.73 m^2^/year		57 (23.2%)		0 (0.0%)		57 (38.0%)	<0.001***

^a^ At the *20-year posttransplant visit*.

^b^ Body mass index.

^c^ ml/min/1.73 m^2^.

^d^ ml/min.

^e^ KDIGO chronic kidney disease classification^25^, according to BSA-deindexed CKD-EPI.

^f^ Protein-to-creatinine ratio (mg/mmol), multiplied by 10.

^g^ According to CKD-EPI, mL/min/1.73 m^2^/year, 15–20 years posttransplant.

### Immunosuppression and DSA-formation

Maintenance immunosuppression is shown in Table 5, results of HLA antibody-screenings in Table 6. Within the first two post-transplant decades, 39/198 (19.7%) KTRs had developed ≥1 DSA, predominantly (29/39, 74.4%) against HLA Class II. Total PIRCHE-II scores (median 42.61 (range: 10.00–111.63) vs. median 33.46 (range: 0.00–99.22)) and PIRCHE-II scores for HLA-class II (HLA-DR; median 11.27 (range: 00.00–58.58) vs. median 4.29 (range: 0.00–28.70)) were significantly higher among KTRs developing DSA compared to KTRs not developing DSA (*p* = 0.021, *p* = 0.020). No differences were observed for PIRCHE-II scores for HLA-class I (HLA-A, -B; median 29.69 (range: 2.07–85.74) vs. median 23.26 (range: 0.00–76.93) between KTRs developing and not developing DSA (*p* = 0.116). Group 1 and group 2 did neither significantly differ in amount of DSA-positive KTRs nor in number, category, or maximal MFI of detected DSA (all *p* > 0.05). No differences were observed for the total PIRCHE-II scores (A, B, DR) and the PIRCHE-II scores per locus between group 1 and group 2 (*p* > 0.05).

**TABLE 5:** Maintenance immunosuppression 20 years posttransplant.

8-year survivors	Total (*n* = 248)		Group 1 (n = 96)		Group 2 (*n* = 152)		*p*-value
	n		n		n		
	248		96		152		
**CNI-based IS**		210 (84.7%)		77 (80.2%)		133 (87.5%)	0.170
**Ciclosporin-based IS**		177 (71.4%)		71 (74.0%)		106 (69.7%)	0.567
CsA/MPA		75 (30.2%)		34 (35.4%)		41 (27.0%)	
CsA/MPA/Steroid		22 (8.9%)		5 (5.2%)		17 (11.2%)	
CsA/Aza		56 (22.6%)		23 (24.0%)		33 (21.7%)	
CsA/Aza/Steroid		14 (5.6%)		5 (5.2%)		9 (5.9%)	
CsA/Steroid		2 (0.8%)		0 (0.0%)		2 (1.3%)	
CsA only		7 (2.8%)		3 (3.1%)		4 (2.6%)	
CsA/mTORi		1 (0.4%)		1 (1.0%)		0 (0.0%)	
**Tacrolimus-based IS**		33 (13.3%)		6 (6.3%)		27 (17.8%)	0.016*
Tac/MPA		17 (6.9%)		4 (4.2%)		13 (8.6%)	
Tac/MPA/Steroid		9 (3.6%)		1 (1.0%)		8 (5.3%)	
Tac/Aza		5 (2.0%)		1 (1.0%)		4 (2.6%)	
Tac/Aza/Steroid		2 (0.8%)		0 (0.0%)		2 (1.3%)	
**mTOR-Inhibitor-based IS (CNI-free)**		12 (4.8%)		4 (4.2%)		8 (5.3%)	0.771
mTORi/MPA		7 (2.8%)		2 (2.1%)		5 (3.3%)	
mTORi/MPA/Steroid		3 (1.2%)		0 (0.0%)		3 (2.0%)	
mTORi/Aza		1 (0.4%)		1 (1.0%)		0 (0.0%)	
mTORi/Aza/Steroid		1 (0.4%)		1 (1.0%)		0 (0.0%)	
**Other**		26 (10.5%)		15 (15.6%)		11 (7.2%)	0.059
Aza/Steroid		19 (7.7%)		11 (11.5%)		8 (5.3%)	
Aza only		1 (0.4%)		1 (1.0%)		0 (0.0%)	
MPA/Steroid		5 (2.0%)		2 (2.1%)		3 (2.0%)	
MPA only		1 (0.4%)		1 (1.0%)		0 (0.0%)	
**Overall Steroid-containing IS**		77 (31.0%)		25 (26.0%)		52 (34.2%)	0.225

IS, Immunosuppression; CNI, calcineurin inhibitor, CsA, Cyclosporine A; MPA, mycophenolic acid, incl. Mycophenolate mofetil, Aza, Azathioprine; mTORi, Mammalian target of rapamycin inhibitor; Tac, Tacrolimus.

**TABLE 6:** DSA screening within the first two posttransplant decades.

20-year survivors	Total (*n* = 248)		Group 1 (*n* = 96)		Group 2 (*n* = 152)		
Excluded		50 (20.2%)		21 (21.9%)		29 (19.1%)	
No Screening^a^		36 (14.5%)		15 (15.6%)		21 (13.8%)	
unknown DSA-onset^b^		14 (5.6%)		6 (6.3%)		8 (5.3%)	
**20-year survivors**	**Total (*n* = 198)**		**Group 1 (*n* = 75)**		**Group 2 (*n* = 123)**		***p*-value**
	n		n		n		
Overall DSA	198	39 (19.7%)	75	11 (14.7%)	123	28 (22.8%)	0.228
HLA-Class I		15 (7.6%)		3 (4.0%)		17 (13.8%)	0.029*
HLA-Class II		29 (14.6%)		8 (10.7%)		26 (21.1%)	0.079
Number of DSA^c^	198	0 (0–6)	75	0 (0–2)	123	0 (0–6)	0.119
Number of DSA^d^	39	1 (1–6)	11	1 (1–2)	28	1 (1–6)	0.062
0	198	159 (80.3%)	75	64 (85.3%)	123	95 (77.2%)	
1	198	27 (13.6%)	75	10 (13.3%)	123	17 (13.8%)	
2	198	8 (4.0%)	75	1 (1.3%)	123	7 (5.7%)	
3	198	1 (0.5%)	75	0 (0.0%)	123	1 (0.8%)	
4	198	2 (1.0%)	75	0 (0.0%)	123	2 (1.6%)	
6	198	1 (0.5%)	75	0 (0.0%)	123	1 (0.8%)	0.512
Number of DSA Class I^c^	198	0 (0–3)	75	0 (0–1)	123	0 (0–3)	0.130
Number of DSA Class I^d^	39	0 (0–3)	11	0 (0–1)	28	0 (0–3)	0.278
0	198	183 (92.4%)	75	72 (96.0%)	123	111 (90.2%)	
1	198	11 (5.6%)	75	3 (4.0%)	123	8 (6.5%)	
2	198	2 (1.0%)	75	0 (0.0%)	123	2 (1.6%)	
3	198	2 (1.0%)	75	0 (0.0%)	123	2 (1.6%)	0.542
Number of DSA Class II^c^	198	0 (0–3)	75	0 (0–2)	123	0 (0–3)	0.191
Number of DSA Class II^d^	39	1 (0–3)	11	1 (0–2)	28	1 (0–3)	0.449
0	198	169 (85.4%)	75	67 (89.3%)	123	102 (82.9%)	
1	198	21 (10.6%)	75	7 (9.3%)	123	14 (11.4%)	
2	198	6 (3.0%)	75	1 (1.3%)	123	5 (4.1%)	
3	198	2 (1.0%)	75	0 (0.0%)	123	2 (1.6%)	0.544
MFI^e^							
Max MFI all DSA	39	5202 (552 21′896)	11	5840 (552–17′203)	28	4765 (681–21′896)	0.618
Max MFI DSA Class I	15	1,146 (552–7,577)	3	653 (552–7,577)	12	1,175 (720–6,278)	0.448
Max MFI DSA Class II	29	6,605 (502–21′896)	8	6,851 (558–17′203)	21	6,605 (502–21′896)	0.981
PIRCHE-II scores							
Total PIRCHE-II score (A, B, DR)	39	42.61 (10.00–111.63)	11	40.80 (14.07–97.72)	28	42.79 (10.00–111.63)	0.852
PIRCHE-II score (A, B)	39	29.69 (2.07–85.74)	11	30.99 (4.31–79.97)	28	28.85 (2.07–85.74)	0.311
PIRCHE-II score (DR)	39	11.27 (0–28.70)	11	11.00 (0–28.70)	28	13.43 (0–58.58)	0.598
DSA-onset (months)^f^	39	211 (0–250)	11	213 (0–250)	28	208 (148–249)	0.492

^a^ No HLA-antibody screening during the observation period.

^b^ First HLA-antibody screening performed beyond 21 years posttransplant and DSA-positive.

^c^ All KTRs.

^d^ DSA-positive KTRs only.

^e^ MFI, mean fluorescence intensity. Highest value measured up to 21 years posttransplant.

^f^ Time from transplantation to first DSA-detection in months.

### KTR-Categorization and Group Comparison

**FIGURE 2:**
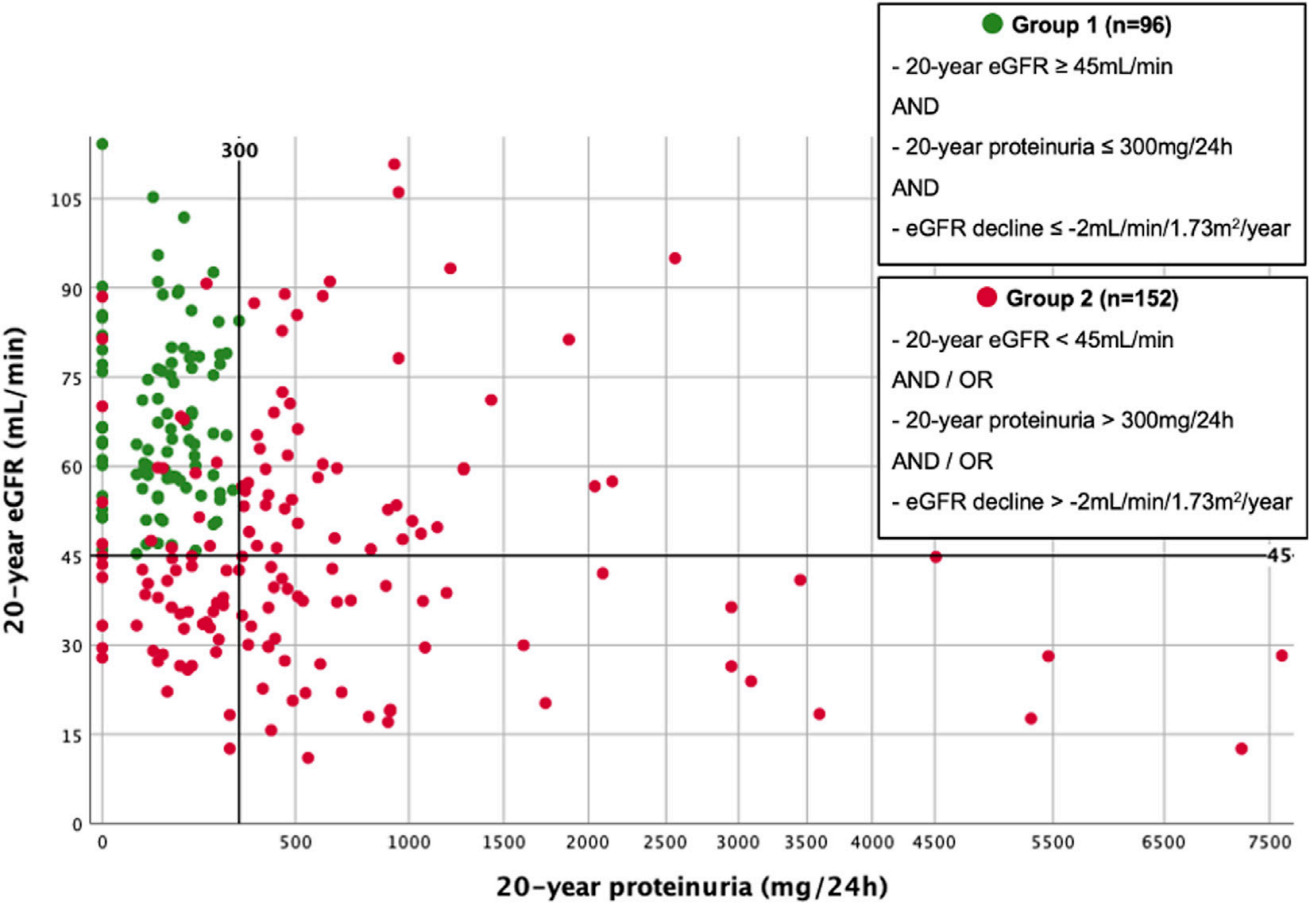
Scatterplot illustrating group subdivision. Scatterplot of all 20-year survivors, according to 20-year eGFR (BSA-deindexed CKD-EPI) and 20-year proteinuria. Group subdivision is marked by color.

Subdivision of the cohort is shown in Figure 1. 96/248 (38.7%) KTRs fulfilled the criteria for superior graft function (group 1). The remaining 152/248 (61.3%) KTRs were classified to group 2. Figure 2 displays distribution of all KTRs according to baseline 20-year eGFR and proteinuria, group subdivision is marked by color.

Multivariable Cox regression analysis is shown in Table 3. The strongest impact on group affiliation was observed for donation after cardiac death (DCD; HR 2.793, 95% CI 1.017–7.667, *p* = 0.041), female sex (HR 2.473, 95% CI 1.329–4.604, *p* = 0.004), early TCMR (HR 2.397, 95% CI 1.222–4.700, *p* = 0.011), and glomerulonephritis as primary disease (HR 2.129, 95% CI 1.152–3.934, *p* = 0.016). While 17 of 152 KTRs (11.2%) of group 2 developed recurrence of primary disease, only 1 of 96 KTRs (1.0%) of group 1 did (*p* < 0.001). A minor impact was observed for donor age (HR 1.037, 95% CI 1.017–1.058, *p* < 0.001) and length of pretransplant dialysis (HR 1.015, 95% CI 1.001–1.030, *p* = 0.041).

### Survival

**FIGURE 3:**
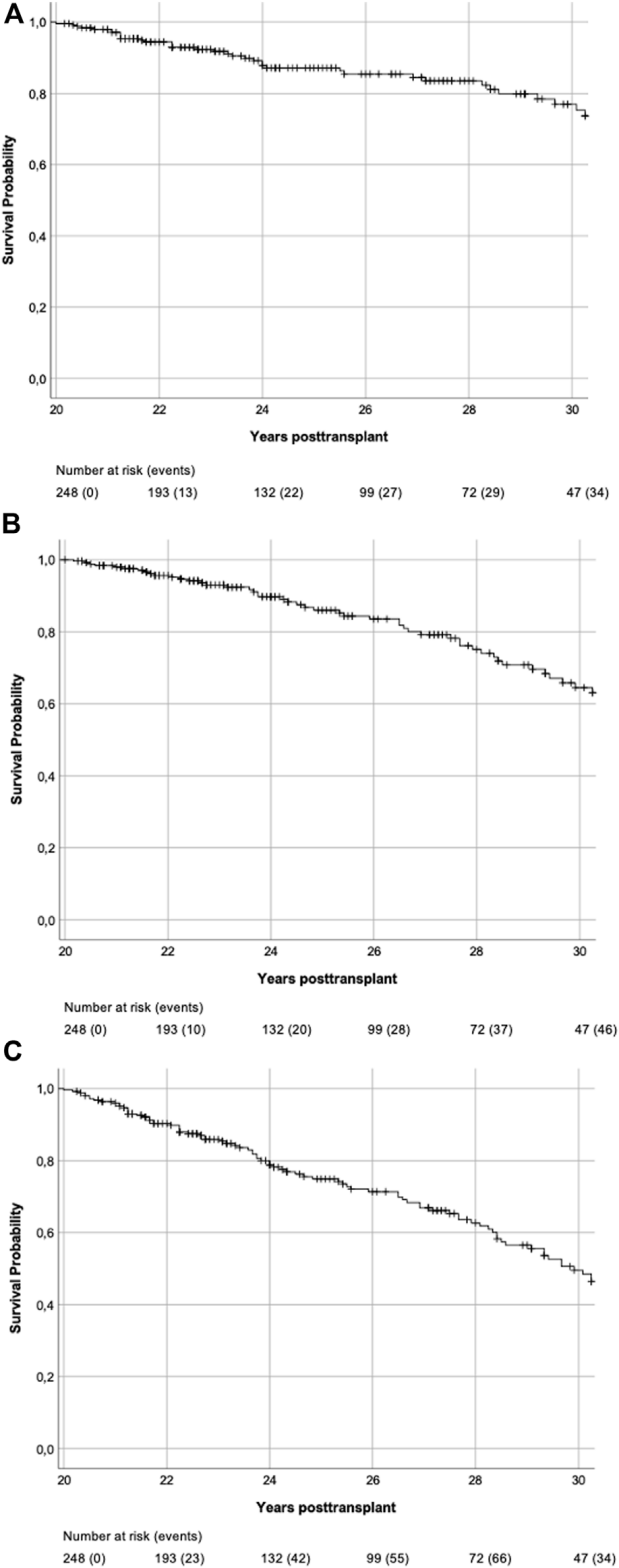
**(A)** Death-censored graft survival. Kaplan-Meier Plot of death-censored graft survival of all 20-year survivors. **(B)** Patient survival. Kaplan-Meier Plot of patient survival of all 20-year survivors. **(C)** Death-uncensored graft survival. Kaplan-Meier Plot of death-uncensored graft survival of all 20-year survivors. Median death-uncensored graft survival was 29.9 years (95% CI 28.4–31.4 years).

**FIGURE 4:**
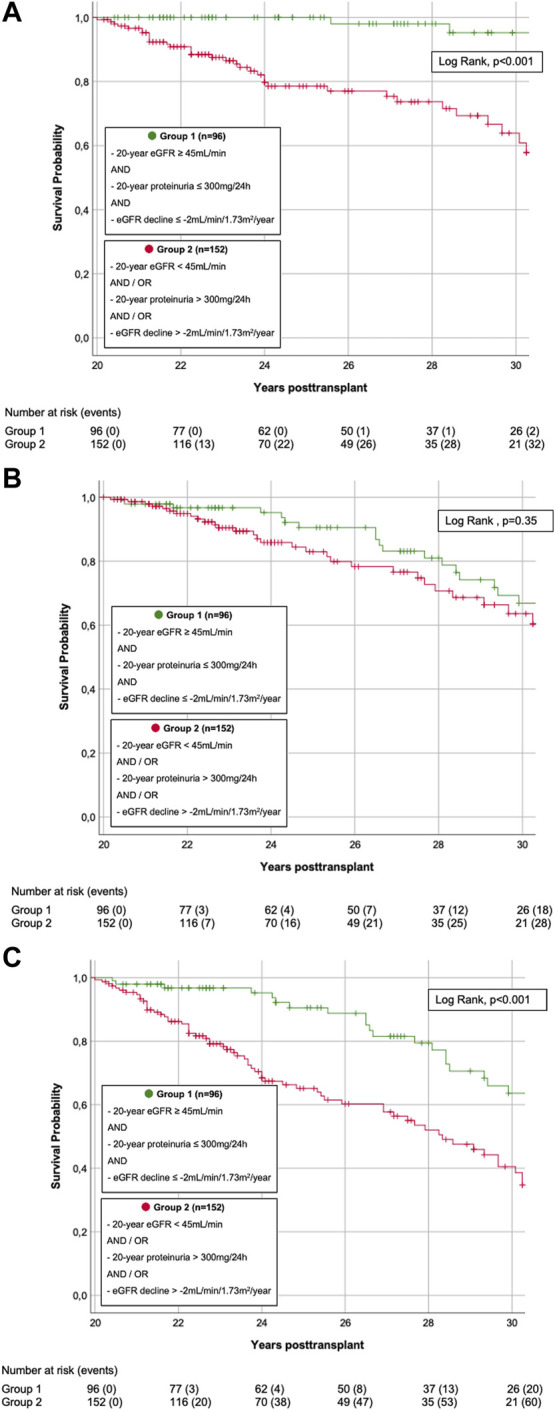
**(A)** Death-censored graft survival. Kaplan-Meier Plot of death-censored graft survival. Death-censored graft survival was significantly superior in KTRs s with superior graft function (group 1) compared to KTRs with inferior graft function (group 2) (LogRank, *p* < 0.001). **(B)** Patient survival. Kaplan-Meier Plot of patient survival. Patient survival did not significantly differ in KTRs with superior graft function (group 1) compared to those with inferior graft function (group 2) (LogRank, *p* = 0.350). **(C)** Death-uncensored graft survival. Kaplan-Meier Plot of death-uncensored graft survival. Death-uncensored graft survival was significantly superior in KTRs s with superior graft function (group 1) compared to KTRs with inferior graft function (group 2) (LogRank, *p* < 0.001).

Survival analyses are shown in Figures 3A–C, 4A–C. 93/248 (37.5%) graft losses were recorded during follow-up: 53/248 (21.4%) KTRs died with a functioning graft (death with functioning graft, DWFG), 40/248 (16.1%) KTRs lost their graft while still alive (death-censored graft failure, DC-GF). Median death-uncensored graft survival was 29.9 years (95% Confidence Interval (CI) 28.4–31.4 years). For death-censored graft survival and for patient survival Kaplan Meier curves did not reach 50%.

In group 1, 26/96 (27.1%) grafts failed during follow-up: 23/26 (88.5%) due to DWFG, 3/26 (11.5%) due to DC-GF. These latter 3 KTRs were analyzed more closely: 1 KTR was DSA-negative 20 years post-transplant but developed *de novo* DSA (dnDSA) during the third post-transplant decade. Graft loss resulted from biopsy-proven chronic antibody mediated rejection (ABMR). The other two KTRs both had their first HLA antibody-screening performed during the 28th year post-transplant and were DSA-positive by then (which, due to indeterminable date of DSA-development, led to exclusion from DSA-sub-analysis, as stated above). Indication biopsy showed glomerulopathy and low level glomerulitis in one, and glomerulopathy and vasculopathy with signs of *de novo* IgA nephropathy in the other case. In group 2, 67/152 (44.1%) grafts failed during follow-up: 30/67 (44.8%) due to DWFG, 37/67 (55.2%) due to DC-GF. Death-censored and death-uncensored graft survival was significantly superior in group 1 (LogRank, both *p* < 0.001, Figures 4A,C). In contrast, there was no significant difference in patient survival (LogRank, *p* = 0.35, Figure 4B).

Univariable and multivariable Cox regression analysis are shown in Tables 7,8. For DC-GF (Table 7), we found a significant impact of group affiliation (HR 20.515, 95%CI 2.730–154.143, *p* = 0.003), overall DSA-development (HR 3.081, 95% CI 1.165–8.146, *p* = 0.023), donor age (HR 1.032, 95% CI 1.004–1.061, *p* = 0.024). For patient survival (Table 8), only KTR-age (HR 1.082, 95% CI 1.051–1.113, *p* < 0.001) and CsA-based immunosuppression (HR 0.297, 95% CI 0.149–0.593, *p* < 0.001) were significantly associated with outcome.

**TABLE 7:** Cox Regression analysis to assess the risk of kidney allograft loss in KTRs 20 years post-transplantation.

Univariate Cox regression	HR	95% CI	*p*-Value
Group (Group 2)	11.533	3.533–37.650	**< 0.001**
20-year eGFR (BSA-deindexed CKD-EPI)	0.926	0.906–0.947)	**< 0.001**
20-year proteinuria	1.001	1.000–1.001	**< 0.001**
eGFR (CKD-EPI) decline	0.786	0.703–0.879	**< 0.001**
Time on pretransplant dialysis (per month)	0.995	0.979–1.012	0.581
Pretransplant dialysis^a^ (PD)	0.664	0.311–1.419	0.291
HLA Mismatch (per mismatch)	0.907	0.686–1.199	0.492
DSA (DSA-positive)	2.719	1.081–6.841	**0.034**
Donation after cardiac death (DCD)	1.684	0.484–5.882	0.413
Donor sex (male)	1.790	0.936–3.424	0.078
Donor Age (per year)	1.043	1.021–1.065	**< 0.001**
Retransplantation (retransplant)	2.094	0.926–4.738	0.076
GN^b^ as the cause of ESKD (all other)	1.463	0.785–2.725	0.231
Transplant era (1981–1989)	0.836	0.414–1.691	0.619
BMI 20 years post-transplant	0.973	0.904–1.047	0.458
HbA1c 20 years post-transplant	1.246	0.770–2.016	0.37
CSA-based immunosuppression (CsA-free)	0.794	0.394–1.598	0.518
steroid-containing immunosuppression (steroids)	2.572	1.382–4.787	**0.003**
Early TCMR (<12 months)	1.847	0.905–3.771	0.092
**Multivariate Cox Regression**			
Group (Group 2)	20.515	2.730–154.143	**0.003**
DSA (DSA-positive)	3.081	1.165–8.146	**0.023**
Donor age (per year)	1.032	1.004–1.061	**0.024**
Steroid-containing immunosuppression (steroids)	2.844	1.295–6.246	**0.009**

^a^ Only HD vs. PD/HD.

^b^ Glomerulonephritis, incl. vasculitis, systemic lupus erythematosus, and suspected chronic GN.

Univariable and multivariable Cox regression models for death-censored graft failure. Reference category in parentheses. HR, hazard ratio; CI, confidence interval.

**TABLE 8:** Cox Regression analysis to assess the mortality risk of KTRs 20 years post-transplantation.

Univariate Cox regression analysis	HR	95% CI	*p*-Value
Group (Group 2)	1.301	0.749–2.261	0.350
20-year eGFR (BSA deindexed CKD-EPI)	0.995	0.981–1.009	0.469
20-year proteinuria	1.000	1.000–1.001	**< 0.001**
eGFR (CKD)-EPI) decline	1.071	0.949–1.208	0.266
Recipinet sex (male)	1.742	1.013–2.995	**0.045**
Recipient age (per year)	1.084	1.054–1.115	**< 0.001**
Time on pretransplant dialysis (per month)	1.003	0.992–1.014	0.616
Pretransplant dialysis^a^ (PD)	0.645	0.335–1.241	0.189
Retransplantation (retransplant)	1.044	0.416–2.625	0.926
GN^2^ as the cause of ESKD (all other)	0.456	0.244–0.854	**0.014**
Transplant era (1981–1989)	1.136	0.613–2.105	0.685
BMI 20 years post-transplant	0.989	0.929–1.053	0.730
HbA1c 20 years post-transplant	1.126	0.727–1.745	0.595
CsA-based immunosuppression (CsA-free)	0.372	0.214–0.645	**< 0.001**
containing steroid-containing immunosuppression (steroid-free)	1.936	1.126–3.327	**0.017**
**Multivariate Cox regression analysis**			
Recipient sex (male)	1.829	1.021–3.276	**0.042**
Recipient age (per year)	1.094	1.062–1.126	**< 0.001**
CSA-based immunosuppression (CsA-free)	0.297	0.149–0.593	**< 0.001**
containing steroid-containing immunosuppression (steroid-free)	1.701	0.866–3.340	0.123
GN^b^ as Cause for ESKD (all other)	0.827	0.429–1.595	0.571

^a^ Only HD vs. PD/HD.

^b^ Glomerulonephritis, incl. vasculitis, systemic lupus erythematosus, and suspected chronic GN.

Univariable and multivariable Cox regression models for patient survival. Reference category in parentheses. HR, hazard ratio; CI, confidence interval.

## Discussion

ULS represent a growing, yet insufficiently studied patient population (8,9). To address this new challenge in transplant long-term aftercare (7,9), we herein analyzed 248 KTRs with a functioning graft ≥20 years. In line with earlier ULS-reports (7,8,9,11,12,14,15) graft function was remarkably good: 20 years post-transplant, the majority (64.5%) of the KTRs was in stage 1-3a of CKD classification (25). 38.7% fulfilled the criteria for superior graft function, i.e., had high and stable 20-year eGFR and low proteinuria (group 1).

Group comparison revealed a significant difference in DCD, early TCMR, recipient gender, and glomerulonephritis as primary disease. For the first, previous studies suggest comparable survival rates for kidneys from DCD. (27) For the second, although in general associated with reduced graft survival, the impact of successfully treated early TCMR on ultralong-term survival has not been well studied. (51) However, our data suggest that initial acute kidney injury and associated nephron loss either due to DCD or early TCMR may have an impact in the ultralong-term, and predispose these KTRs to decreased graft function and proteinuria through chronic hyperfiltration and chronic histologic lesions of interstitial fibrosis/tubular atrophy. Regarding the effect of KTR-gender on ultralong-term survival, results are conflicting (8,9,10,13). However, the predominance of men in group 1 surprises, given their higher risk of chronic graft failure (28). Our finding could result from potential underestimation of GFR in women by the applied equations and stresses the need for further, gender-specific studies (29). Although the time of onset and severity of recurrence of the underlying disease vary widely, our data suggest that glomerulonephritis recurrence strongly influences the risk of impaired renal function in the ultralong-term. However, in our analysis, no factor had an independent impact on further survival.

Data on long-term maintenance immunosuppression is extremely limited (30). In our center, standard immunosuppression during the respected period was composed of Ciclosporin, Azathioprine and Corticosteroids. 20 years post-transplant, 71% of the ULS were still under Ciclosporin-based maintenance therapy (no significant group difference). However, group 2 contained significantly more KTRs with Tacrolimus-based immunosuppression. Changes in immunosuppressive therapy over time could not be analyzed in this study, but we presume that this difference results from conversion from Ciclosporin to Tacrolimus in response to supposed immune-related injury and the development of DSA by intensifying maintenance immunosuppression (30). The deleterious effect of calcineurin inhibitors (CNI) on long-term graft outcome has become an increasing matter of debate (31,32). Regarding ULS, data is scarce and inconsistent: Bererhi et al. only found 3% of ULS with CNI-based maintenance immunosuppression and therefore hypothesized that avoiding CNI could favor ultralong-term graft survival (7). In contrast, Traynor et al. reported 40% and Kettler et al. even 68% of ULS with CNI-based therapy (8,12). Given the prolonged exposure to immunosuppression, determining optimal long-term immunosuppression is especially important for ULS (9). But this urgent question still remains unanswered (33).

To target therapeutic interventions and optimize ultralong-term aftercare, we need to improve our understanding of late graft loss (16,31), which includes patient’s death (DWFG) and loss of graft function while still alive (DC-GF) (16). In this study, we drew a detailed picture of graft and patient survival of 248 ULS. While overall graft survival was already remarkably good (median death-uncensored graft survival 29.9 years), for KTRs with superior graft function, it was outstanding. In fact, group 1 only involved 3 events of DC-GF. In contrast, graft survival in group 2 was clearly inferior. Corresponding with the fact that graft failure is preceded by graft dysfunction (16,34), 92.5% of all events of DC-GF in this study occurred in group 2.

In their comprehensive study of 177 ULS, McCaughan et al. observed that DC-GF after 20 years is uncommon (9). Our study shows that this is particularly true for ULS with preserved graft function, while in group 2, DC-GF accounted for the majority (55.2%) of graft losses. A multivariable Cox regression model for DC-GF confirmed a strong influence of group affiliation.

Donor age profoundly impacts graft quality (35) and is an important risk factor in graft outcome (2,35). In this study, comparable to earlier ULS-reports (7,8,9,10,12,13), donors were young (median 32 years), a clear difference to more recently transplanted KTRs (2). Very interestingly, donors were significantly younger in group 1, and donor age had significant impact on DC-GF beyond 20 years post-transplant. This phenomenon might be attributed to the loss of functional nephrons with aging and consecutive decreased functional reserve (36) and increased vulnerability to transplant-related injury (37,38). In previous studies, univariable analyses revealed significant association of donor age with ultralong-term survival, however, in multivariable models the effect showed only a trend and missed statistical significance (9,10).

Late DC-GF is profoundly driven by alloimmune mechanisms (31,39,51). DSA are associated with increased risk of late graft failure (40,41) and provide a well-established biomarker predicting ABMR and graft loss (40,42). However, little is known about the role of DSA in the context of ultralong-term survival (12,43). Analysis of DSA-screenings revealed several interesting findings: First, in our cohort of ULS only, cumulative incidence of DSA-formation during the first two post-transplant decades was 19.7% and thus within the range reported from general KTR-population (42,44). Secondly, we could not find any significant group difference in cumulative incidence of DSA-positive KTRs, duration to first DSA-detection, HLA-class, HLA-mismatches, PIRCHE-II socres, MFI, and number of detected DSA. These results surprise, as KTRs who develop dnDSA have been shown to have higher rates of eGFR decline (41). Thirdly, however, DSA-formation was identified as an independent risk factor for DC-GF. The association of steroid use with DC-GF must be interpreted here with the restart of steroids after the onset of DSA. In our cohort, DSA were detected surprisingly late (median 211 months post-transplant), a finding probably biased by transplant era and available techniques. However, it is known that DSA-formation can appear anytime, even several years post-transplant (42,43,44) and that time from dnDSA-onset to graft dysfunction ranges from months to years (44). So nevertheless, it is suggestive that ultralong-term survival of our cohort was favored by substantially late DSA-development and that their deleterious impact on graft survival manifested not until the third post-transplant decade. Given the close relationship between dnDSA, ABMR and ultimate graft loss, this result points towards a potential target of intervention in order to further improve long-term graft survival (45). However, further studies are needed to address this question (45).

Hence, DC-GF is predominantly seen in ULS with inferior graft function. However, despite the known link of declining graft function with increased mortality (46), there was no significant group difference in patient survival. The risk of DWFG increases with time since transplantation (47) and in ULS, it represents the leading cause of graft loss (8,9,14,15). Our results correspond with the findings from Gaston et al. who stated that mortality risk is largely independent of graft function (16). Beyond 20 years post-transplant, leading causes of death are cardiovascular disease and malignancy (8,9), both highly prevalent in ULS (7,8,9,14,15). For example, McCaughan et al. reported cancer in 37% and cardiovascular disease in 27% of 20-year survivors and therefore stated that, in management of ULS, focus should shift on prevention and optimal therapy of these comorbidities (9).

Our results allow us to specify this statement and lead to further clinical implications. Indeed, in case of good, stable graft function up to 20 years post-transplant, risk of ultimate DC-GF is very low, and focus should be on controlling the medical comorbidities (9). In contrast, in KTRs with inferior ultralong-term graft function, risk of DC-GF may not be neglected, and aftercare should equally concentrate on preventing ultimate loss of graft function. Given that DSA and ABMR, respectively, are potentially treatable conditions (45). Our findings argue for continuing DSA-monitoring even in the setting of ultralong-term survival. Additionally, KTRs with inferior ultralong-term graft function might be considered for biopsy, not only to evaluate immune injury, but also to detect evidence of CNI-toxicity and to adjust immunosuppression, accordingly (48,49).

Our study has several limitations. First, it is a retrospective and single-center analysis with the intrinsic limitations and potential biases. Secondly, our cohort differs in several aspects from more recently transplanted KTRs. Thirdly, DSA-subanalysis is limited by transplant era and available techniques: it affected time of first and frequency of subsequent screenings, 50 cases with missing or insufficient data had to be excluded, and KTRs were mostly not typed for HLA-DP and -DQ.

However, most of these limitations are inevitably associated with the retrospective design of research on ULS and the according necessity of lengthy follow-up (8). In addition, our study cohort is not dominated by living donor transplantations, which would suggest better organ quality and possibly better HLA matching, so our results translate well to the general transplant cohort. The reason for this is that only about 50 living donations were performed in the observation period from 1981 to 1999 at our center. Our study provides an important contribution in improving understanding of this unique, increasingly important patient population (7,8,9). Comprehensive follow-up enables us to give extensive overview of ultralong-term graft function, alloimmunization, and ultimate outcome beyond 20 years post-transplant and to identify corresponding risk factors and potential therapeutic targets required to improve ULS-aftercare.

## Conclusion

Overall, KTRs with ultralong-term survival ≥20 years do extremely well. Particularly KTRs with stable and high eGFR and low proteinuria likely keep their graft function and ultimately die of medical comorbidities. The risk of graft failure is predominantly seen in KTRs with inferior graft function. This graft function-related risk profile could augment long-term monitoring and treatment.

## Data Availability

The data underlying this article will be shared on reasonable request to the corresponding author.

## Abbreviations

ABMR, Antibody-mediated rejection; BMI, Body mass index; BSA, Body surface area; CI, Confidence interval; CKD, Chronic kidney disease; CKD-EPI, Chronic kidney disease epidemiology collaboration; CNI, Calcineurin inhibitor; CTS, Collaborative Transplant Study; DCD, Donation after cardiac death; DGF, Delayed graft function; DSA, Donor specific antibody; dnDSA, *De novo* donor specific antibody; DC-GF, Death-censored graft failure; DWFG, Death with functioning graft; eGFR, Estimated glomerular filtration rate; ESKD, End stage kidney disease; HLA, Human leukocyte antigen; KTR, Kidney transplant recipient; MDRD, Modification of diet in renal disease; MFI, Mean fluorescence intensity; PCR, Protein-to-creatinine ratio; ULS, Ultralong-term survivors.
