# Supplementary material for: Entering the Third Decade After Kidney Transplantation: Excellent Graft Function Refers to Superior Graft but Not Patient Survival
Source: Transpl Int. 2022 Oct 31;35:10675. doi: 10.3389/ti.2022.10675 (PMC9659610; doi:10.3389/ti.2022.10675)
Supplement: Supplementary file 1 [file Table1.docx]

**Supplement Table 1. CKD-related laboratory findings 20 years post-transplant**

| **20-year survivors** |  | **Total (n=248)** | |  | **Group 1 (n=96)** | |  | **Group 2 (n=152)** | | **p-value** |
| --- | --- | --- | --- | --- | --- | --- | --- | --- | --- | --- |
|  | n |  |  | n |  |  | n |  |  |  |
| Hb g/L | 247 | 125 | (80-163) | 96 | 131 | (90-163) | 151 | 119 | (80-161) | **<0.001***** |
| 25-OH VitD μg/L | 153 | 26.9 | (3.8-66.9) | 56 | 27.2 | (5.3-66.9) | 97 | 26.9 | (3.8-58.4) | 0.938 |
| PTH ng/L | 211 | 73.4 | (6-742.1) | 76 | 63.2 | (10-190) | 135 | 82.4 | (6-742.1) | **0.001**** |
| HCO3- mmol/L | 215 | 23 | (10-32) | 83 | 24 | (18-32) | 132 | 22 | (10-29) | **<0.001***** |
| LDL mmol/L | 209 | 2.5 | (0.7-5.6) | 78 | 2.5 | (0.8-4.8) | 131 | 2.6 | (0.7-5.6) | 0.388 |

Hb = Hemoglobin. 25-OH Vit D = 25-OH Vitamin D. PTH = Parathormone. HCO3- = Bicarbonate. LDL = low density lipoprotein cholesterin.
